# Medical and Philosophical Intelligence

**Published:** 1813-02

**Authors:** 


### MEDICAL AND PHILOSOPHICAL INTELLIGENCE.

### *ADDRESS of the COMMITTEE of APOTHECARIES.*

AS the period is now fast approaching at which the intended Bill for the improvement and melioration of the situation of the General Medical Practitioner must be presented to parliament, in order to secure its consideration during this session, the Committee conceive it incumbent to refer to the profession the following, as the outline of the principal topics which the bill is intended to embrace. They respectfully solicit the opinions both of individuals and of district meetings on the subject, to direct their judgment in adapting the more detailed parts to the circumstances of practitioners in every part of the country.

The profession have a very apposite precedent in the act which passed the Irish parliament in 1791, granting considerable powers and privileges for the protection and regulation of the Apothecaries throughout the whole kingdom of Ireland. But the advantageous consequences that have resulted from the regulations which were enjoined by the legislature, some years since, on the practice of the attorney, presents convincing evidence that numerous evils may be corrected, and an honorable and useful profession rescued from obloquy, by judicious and well-applied laws. The Committee, therefore, have decided—1st, that requiring a suitable education is the surest barrier against the intrusion of ignorant practitioners and pretenders; 2dly, that the imposing of a stamp-duty of 25*l.* on apprentices' indentures, will prevent the introduction of individuals of too

 inferior inferior rank: and 3dly, that, by paying a trifling sum for an annual licence, (which will be registered and published,) the public will always have the power of distinguishing the qualified practitioner from the pretender; while the former will enjoy the fair and undisturbed reward of his professional abilities and exertions.

By establishing a legal but optional claim for attendance, in cases of midwifery, and visits or journeys, the general practitioner will have a right confirmed which he does not at present possess.

The Committee lament that their Report, and the Resolutions of the General Meetings of Apothecaries, have been so partially distributed; which has arisen from the want of an accurate register of the names and residences of country practitioners. This defect it is now of the utmost consequence immediately to correct. It is, therefore, earnestly requested that gentlemen should take some pains to obtain precise information of the numbers of every description of medical practitioners, whether regular or irregular, in their county; which, with the lists of provincial medical societies, and any other communication that will promote the intentions of the Committee, must, without further delay, be forwarded to London.

The Committee appointed a deputation of their members, consisting of Mr. Brande, Apothecary to the King, Mr. Upton, Mr. A. T. Thomson, and the Chairman, (being two apothecaries and two surgeon-apothecaries,) to wait upon the Chancellor of the Exchequer, to submit to him the heads of their proposed plan. The Chairman has since been honored with another interview. On both occasions, Mr. Vansittart's comprehensive mind appeared, at once, to be fully impressed with the utility of the measure, and the important benefits both the public and the profession would derive from its immediate adoption: and, to facilitate this national desideratum, he condescendingly wrote to His Majesty's Post-master General, requesting that an arrangement might be settled through which the correspondence could be conducted free of expense.*

If proofs were wanting of the necessity of the regulations contemplated by the Committee, it would be only to publish the accounts of the zeal and alacrity with which meetings have been held in the counties of Middlesex, Kent, Surrey, Sussex, Hants, Dorset, Wilts, Somerset, Devon, Salop, Worcester, Warwick, Northampton, York, Lancaster, Derby, Lincoln, Cambridge, Huntingdon, Bedford, Bucks, Berks, Herts, Essex, Suffolk, Norfolk, &c. &c. where resolutions have been passed, declaring their determination to call the attention and support of their representatives to the bill in its progress through

* It having been announced that this accommodation would interfere too much with the regulations of the Post Office, Mr. Vansittart has since intimated to the Chairman, that a few of the packets going to distant parts of the country shall be dispatched in official franks: a favor which will afford at least the opportunity, through the post-masters of many populous places, of applying to the medical practitioners with whose names the Committee regret they are so entirely unacquainted.

parliament, parliament. They likewise sent copies of their resolutions, a practice much to be commended, to the contiguous districts; and have been most laudably prompt and liberal in their subscriptions.

The Committee have much satisfaction in stating, that the funds are progressively increasing; but, as a sufficient sum must be obtained to meet every contingency, before the petition is presented to parliament on the 2d of February, or the bill is carried into the House, they must impress upon every individual practitioner the obligation of immediately contributing in aid of the subscription. For, though no opposition can be reasonably anticipated, and they have the strongest grounds to expect that their exertions will terminate in success; yet, if any should unfortunately occur, the means of meeting it must be previously insured. With this in view, it becomes absolutely expedient that single subscriptions be directly transmitted to London; and the amount of collective subscriptions already on the books of district meetings be made known to the Chairman of the Committee, that a measure so beneficial to the public and the medical profession may not be defeated and lost.

The Committee pledge themselves that a system of œconomy, consistent with the active prosecution and ultimate attainment of their plan, shall be exercised, of which subscribers will have full opportunity of judging. If a surplus fortunately remain, there are many professional and useful purposes to which it may be appropriated, that would afford eminently superior advantages to future students in the principles and practice of medicine, and reflect lasting honor on the character, liberality, and enlightened prospective views, of the surgeons and apothecaries of the present epoch.

*Outlines of a Plan proposed by the London Committee, for improving and protecting the Profession of the Apothecary, Surgeon-Apothecary, and Practitioner in Midwifery, in England and Wales.*

I. The provisions of the proposed bill, so far as relates to qualifications, will be prospective, and therefore not applicable to persons already in practice.

II. No person to be allowed to enter the profession without having served a regular apprenticeship, or producing testimonials of a suitable medical education.

III. No person to be allowed to practise as apothecary, surgeon-apothecary, or accoucheur, without obtaining a certificate after due examination of his qualifications.

IV. No person to be allowed to practise in any branch of the profession, or to compound or dispense medicines, without taking out an annual license, which is proposed not to exceed 2*l.*

V. Every apprentice's indenture to bear a stamp duty, not exceeding 25*l.*

VI. A superintending body to be legally constituted for the several objects of the proposed act, with power to make such by-laws and regulations as may be necessary to carry them into effect.

VII. England and Wales is to be divided into a certain number of medical districts, of which London shall be the superior, and where only certificates for qualifications can be obtained.

VIII.

VIII. Assistants to undergo an examination.

IX. There shall be a legal right for apothecaries, surgeon-apothecaries, and accoucheurs, to claim a moderate charge for their attendance, as visits or journeys.

X. The present improper system of remuneration for medical attendance on the parochial poor, requiring immediate regulation, early communication on this subject from provincial practitioners will be particularly necessary.

XI. A register to be established, in every district, of all licensed practitioners, apprentices, &c.

*Bloomsbury-square,* G. M. Burrows,
*Jan.* 22, 1813. Chairman of the Committee.

The Committee beg leave to inform the Profession that the Petition will lie at the Crown and Anchor Tavern, Strand, from January 28th to February 2d, for signatures; and where also Subscriptions may be paid; or to the Chairman; Mr. Simons, or Mr. Field, the Treasurers; or at Messrs. Goslings and Sharpe, Bankers, No. 19, Fleet-street.

SUBSCRIBERS.

Acret, Francis-street
Atkinson, Jermyn-street
Andrews, Charing Cross
Ansell and Curtis, Dorking, Surry
Adams, Charlotte-street, Fitzroy-square
Austin, Charles-street, Northampton-square
Bowman, Harley-street
Bamford, Conduit-street
Brown, Raven-row, Spitalfields
Blatch, Bath-place, New Road
Blieth, Great Russel-street
Burrows, Bloomsbury-square
Brand, A. E. Chiswick
Brand, E. A. Arlington-street
Brand, William, ditto
Burrows, Bishopsgate-street
Browne, Oxford-street
Burchell, Harley-street
Broxholm, Sunbury, Middlesex
Briden, Great Coram-street
Best, Tavistock-street
Bunnett and Son, Fulham
Bethune, Brighton, Sussex
Bent and Lerew, Great Portland-street
Burman and Burt, Diss, Norfolk
Birdsall, Pickering, Yorkshire
Benson, Goswell-street-road
Bowes, Richmond, Surry
Brougham, Finsbury-place
Cooke, Plaistow, Essex
Carruthers, Tunbridge, Kent
Carruthers, Mile-end
Cates, Argyle-street
Chilver and Tupper, New Burlington-street
Chapman, Wandsworth
Carolan, Charlotte-street, Fitzroy-square
Carolan, 151, Fleet-street
Casue, Bedford-street, Bedford-square
Cole, Russel-street, Covent Garden
Chamberlain, Aylesbury-street, Clerkenwell
Coombe, Caroline-street, Bedford-square
Clapton, King-street, Westminster
Coleman, Professor, Veterinary College
Desormeaux, Barking, Essex
Damant and Dale, Hatton Garden
Douglass, Bedford-square
De Bruyn, North Audley-street
Drew, Gower-street
Day, Isleworth
Dunston, Old Broad-street
Davis, Holborn Hill
Dyer, Woodbridge, Suffolk
Egleton, Lisson-street, Paddington
Ewbank, Upper Grosvenor-street
Edwards, Putney
Field and Son, Newgate-street

Forbes,

Freake, Tottenham Court Road
Forbes, New-street, Hanover-square
Fernandez, New Ormond-street
Fincham, St. Alban's-street
Fallofield, Albemarle-street
Fenick, Manchester-street
Frost, Kelvedon, Essex
Fortescue and Barnet, St. John-street
Forster, Oswald Hitchen, Hants
Gilson, Wood-street, Spitalfields
Good, Guilford-street
Gardiner, Clapham
Griffinhoofe and Sheppard, Hampton
Griffiths and Son, Tottenham-court-road
Green, Manchester-street
Gillman, Highgate
Grice, Kensington Gravel-pits
Graham, Chelsea College
Griffiths and Son, Leadenhall-street
Humby, St. Alban's-street
Hayes, Cleveland-street
Hobson, Great Mary-le-bone-street
Hill, Trinity-square
Hunter, J. Mincing-lane
Hunter, J. jun. ditto
Hunter, T. ditto
Harkness, Broad-street, Ratcliffe
Hill, Bedford-row
Hardwicke, Epsom, Surry
Hurlock, St. Paul's Church-yard
Hulke, Deal, Kent
Harton, Pall Mall
Hingeston, Cheapside
Hayman, Axminster, Devon
Hurst, Strand
Harrison, Thayer-street
Hailes, Hoxton
Halmestead, Barking, Essex
Harrison, Braintree, Essex
Hall, Southampton-row
Hunt, Dartmouth, Devon
Jones, Prichard, and Sheldon, Mount-street
Julius, Richmond, Surry
Jones, Chancery-lane
Jackson, Craig's-court, Charing Cross
Johnston, Mortimer-street
Isaacson & Webb, Goodge-street
Jackson, Bishop's-court, Chancery-lane
Ireland and Moore, Barking and Ilford, Essex
James, Croydon, Surry
Jukes and Walton, Stourport, Worcester
Johnston, Wallbrook
Kerrison, New Burlington-street
Kennard, New Bond-street
Kiernan, Charlotte-street, Bloomsbury
King, Mortlake, Surry
Kilby, Watford, Herts
Kilpatrick, St. Martin's-lane
Leuthwaite, Devonshire-street
Lewis, Brunswick-square
Lloyd, Tring, Herts
Leese, Baker-street
Lincoln, Hatton Garden
Lock, Debenham, Norfolk
Leese, E. Finsbury-square
Lomax, Dorset-street
Lucas, Hatfield, Herts
Merrison, Chelsea
Malim, Carey-street
Moss, Somers' Town
Mathias, ditto
Mackinder, Paddington
M'Donald and Blundell, Chiswell-street
Mainwaring and Jones, Strand
Morgan, Bedford-row
Macann, Parliament-street
M'Nab, St. Martin's-lane
Millington, 12, Mortimer-street
Mogg, Tunbridge Wells
Macfarlane, Devizes, Wilts
May, George, Barking, Essex
Newington, Bishopsgate-street
Newnham, Farnham, Surry
Nevinson, C. esq. Saville-row
Newsome, J. esq. Islington
North, Chelsea
North, Molyneaux-street
Owen, Chancery-lane
Ogilvy, H. Southampton-buildings
Oyle, Great Russel street
Owen, Little Britain
Parkinson, Hoxton-square
Powel, Great Russel-street
Parrott, Mitcham, Surry
Pilliner, Dartmouth-street
Peregrine, Half-Moon street
Parslow, Pimlico
Pennington and James, Lamb's Conduit-street
Paumier, Watford, Herts

Pout,

Pout, Yaldins, Kent
Pharmacopolus Verus
Perico, South Molton-street
Russell, jun. Mount-street
Robinson, Great Portland-street
Rivers and Freeman, Spring Gardens
Ring, Park-street
Robins, Tottenham Court-road
Radford, R. esq. Hammersmith
Revans, Vauxhall
Robinson, Cooper's-row
Robinson, Doughty street
Royston, Princes'-street, Cavendish-square
Rumsey, Amersham, Bucks
Roberts, Hatton Garden
Roper, Bunhill-row
Simpson, Harpur-street
Shillito, Putney
Sharpe, Tooting, Surry
Stuart, Great Mary-le-bone-street
Soley, Hart-street, Bloomsbury
Simons and Watson, Soho-square
Starr, Smithfield Bars
Smith, Upper Berkeley-street, Portman-square
Simmons, Axendon-street
Smith, Great Portland-street
Smith, Smith-street, Chelsea
Sutherland & Annandale, Queen-street, Westminster
Seaton, Bridge-street, Westminster
Sutton, Town, Mallins
Stewart, Hatfield, Herts
Seymour, Bishop's Waltham, Hants
Sharpe, Hoxton-square
Steele, Tower Hill
Spry, Charter-house-square
Thompson, Sloane-street
Thompson, Stockwell
Thompson, Kensington
Taynton, Bromley, Kent
Upton, Throgmorton-street
Wheeler, St. Bartholomew's Hospital
Whittle, Mount-street
Wells and Wells, Welbeck-street
Ward, Holles-street
Winkfield, Market-street
Welbank, Chancery-lane
Wells and Eyles, Ely-place
Walker, St. James's-street
Wright, Grenville-street
Wallace, Carshalton, Surry
Wood, Edgeware-road
Weston, Shoreditch
Wilkinson, Long Sutton, Lincolnshire
Wheeler, W. esq. Ludgate-hill
Wood, Princes'-street, Hanover-square
Wood, Great Titchfield-street
Williams, Vere street, Cavendish-square
Weeks and Son, Hurst-le-point, Sussex
Willis, Bow, Essex
Ward, Curtain-road
Westrop, Leonard-street
Walker, Ongar, Essex
Whicher, Petworth, Sussex
Wynne, Robert, Liverpool
Young, Lambeth
Yonge, Bennet-street, St. James's.

*Hampstead, Middlesex.*
By John Bliss, esq.

Messrs. Bliss, Haines, and Hogg, Hampstead and Hendon
Mr. Jacobs, Hampstead
Mr. Heathcot, ditto
Mr. Rodd, ditto

*Marlborough, Wiltshire.*
By T. Maurice, esq.

T. Maurice, Marlborough
N. Washbourn, ditto
R. Hooper, Wootton Bassett
W. Bartlett, Great Bedwin
R. Somersett, Marlborough
J. Gay, Swindon
Tho. Richens, Barbage
G. Barnes, Pewtsey
J. Tarrant, Swindon
W. Gay, Highworth
R. Barker, Hungerford
R. K. Marsh, Ramsbury
J. Somersett, Lambourn
Tho. Major, Hungerford

*Basingstoke, Hampshire.*
By C. Covey, esq.

C. Newnham, Alton
W. Curtis, ditto
W. Seward, ditto
J. Workman, Basingstoke
Cha. Lyfford, ditto
C. Covey, ditto
C. Howard, Hartley-row
J. Jackson, Hartfield
C. Shebbeare, Odiham

G. Cambden,

G. Cambden, Odiham

G. Leach, ditto

W. H. Ledbeater, Overton

—— Hunter, Whitchurch

*Beccles, Suffolk.*

By W. Henchman Crowfoot, esq.

W. M. Crowfoot, Beccles

W. H. Crowfoot, ditto

H. S. Davey, ditto

C. Dashwood, ditto

R. Camel, Bungay

L. L. Davie, ditto

*Modbury, Devon.*

By Wm. Langworthy, esq.
Wm. Langworthy, Modbury
G. T. Langworthy, ditto
W. S. Langworthy, ditto

*Dover, Kent.*

By F. Thatcher, esq.
F. Thatcher, Dover
Edw. Norwood, ditto
P. Elsted, ditto
Stephen Chalk, ditto

*Wisbech Medical Society.*

By W. Skrimshire, jun. esq.
Alex. Fraser, M.D. Wisbech
Fenwick Skrimshire, M.D. Peterborough
Rob. Hardwicke, esq. Wisbech
W. Archbould, ditto
W. Skrimshire, jun. ditto
Dawes, ditto
Stear, ditto
Weatherhead, ditto
Herring, ditto
J. Johnson, ditto
Wales, Downham
Wray, March
Corthorn, ditto
Bell, St. John's
Bamford, Holbech
Bailey, Long Sutton
Webb, Upwell

*Newport, Salop.*

By J. E. Stannion, esq.
T. Baddeley, sen. Newport
T. Baddeley, jun. ditto
Rob. Hughes, ditto
J. E. Stannion, ditto
R. Higgins, ditto

*Milford, Suffolk.*

By R. Anderson, esq.
Edwards, Milford
Ross, Sudbury
Duddle, Bury
White, Clare
Murray, Sudbury
Anderson, ditto

*Halifax, Yorkshire.*

—— Alexander, esq. in the Chair.
Resolutions received, but not the names of the Gentlemen present.

*Rotherhithe, Surrey.*

By Wm. Gaitskell, esq.
W. Gaitskell, Rotherhithe
Brown and Randall, ditto
John Ford, ditto
Rich. Wright, ditto

*Wellington, Somerset.*

By Humphrey Langley, esq.
Andrew Holman, Wellington
F. S. Bridge, ditto
H Langley, ditto
Peter Goullett, ditto

*Walsall, Warwickshire.*

By Tho. Adams, esq.
J. Wollatt, Walsall
F. W. Weaver, ditto
J. Parnell, ditto
E. Elwell, ditto
Tho. Pitt, ditto
Tho. Adams, ditto

*Reigate, Surrey.*

By Tho. Martin, esq.
Smith, Crawley
Steele, Reigate
T. Martin, ditto

*Rochester, Kent.*

By Cha. Thompson, esq.
Richard Thompson, esq. Rochester
C. Thompson, ditto
W. Adams, ditto
R. Fry, ditto
A. Rye, ditto
G. G. Watkins, ditto
C. Bathurst, Stroud
E. Edwards, ditto
Tho. Hope, Chatham
J. Bryant, ditto
W. Witheridge, ditto
R. Martin, ditto
J. Ely, ditto
W. Godfrey, ditto
R. Morris, Brompton
P. Cullen, Sheerness
T. Coleman, ditto
W. Castle, Sittingbourne
G. M. Tracey, ditto
Nice, Milton
W. H. Rogers, Gravesend

*Romford, Essex.*

Carruthers, Romford

Andrews, ditto

Davis, ditto

*Northampton.*
By H. Locock, esq.
Clark, Northampton
Locock, ditto
Thomas, ditto
Cooke, ditto
Hall, ditto
Percival, ditto
Osborn, ditto
Rudsell, ditto

*Rye, Sussex.*

By James Bellingham, esq.

J. Bellingham, Rye

Wm. Ramsden, ditto

R. W. Butler, ditto

Satterley, Hastings

John Wellings, ditto *

Henry Sutton, Winchelsea

Edw. Young, Hawkshurst, Kent

Walter, New Romney

Willmanton, ditto

Lewis, ditto

*Havant, Hampshire.*
By John Hicks, esq.
Resolutions received, but not the names of the Gentlemen present.

*Stourbridge, Worcester.*
By Wm. Evans, esq.
J. Causer, Stourbridge
W. Evans, ditto
Edw. Dixon, ditto
J. Causer, jun. ditto
W. Freer, ditto

---

*Royal Society.*—Saturday the 30th November, being St. Andrew's day, the Royal Society held their annual meeting, at their apartments in Somerset-place, for the choice of a council and officers for the year ensuing, when the following gentlemen were elected:

Of the old council:—The Right Hon. Sir Joseph Banks, bart. K.B.; Sir Charles Blagden, knt.; Samuel Goodenough, Lord Bishop of Carlisle; Anthony Carlisle, esq.; Sir Humphry Davy, knt.; Samuel Lysons, esq.; Joseph de Mendoza Rios, esq.; George Earl of Morton; John Pond, esq. astronomer royal; William Hyde Wollaston, M.D.; and Thomas Young, M.D.

Of the new council:—George Earl of Aberdeen; Taylor Combe, esq.; Sir Thomas Frankland, bart.; Silvester Lord Glenbervie; Philip Earl of Hardwicke; Matthew Raper, esq.; Samuel Rogers, esq.; Smithson Tennant, esq.; Rev. William Tooke; and Roger Wilbraham, esq.

Officers.—The Right Hon. Sir Joseph Banks, bart. K.B. president; Samuel Lysons, esq. treasurer; William Hyde Wollaston, M.D. and Taylor Combe, esq. secretaries.

Dec. 10.—The society again assembled, the Right Hon. Sir Joseph Banks, bart. president, in the chair, when a paper, by Mr. Smithson, on ulmin, was read. Mr. S. was so fortunate as to procure a specimen from the same gentleman, at Palermo, who originally forwarded it to Klaproth, and consequently operated on a substance perfectly similar to that of its original discoverer. The author found that the characters given to the substance called ulmin by Dr. T. Thomson, are very incorrect; and, instead of its being a resinous substance, he found that it chiefly consisted of extractive matter united with potash. It was very slightly soluble in alcohol. Mr. S. made some experiments on the juice and branches of English elm, which he found somewhat different from the substance sent him

---

* The accounts of County and District Meetings have multiplied to an extent that compels us to defer their insertion, in part, till our next publication.—Editors.

from from Sicily. He did not, however, pursue his experiments so far as to determine the exact nature of the matter obtained from the native tree.

Dec. 17.—Dr. Wollaston, availing himself of the facts reduced to practice by Leslie, in freezing water by means of evaporation in vacuo, described an instrument with which this process might be effected for amusement. Taking a glass tube of any length, such as used for barometers, making a bulb on each end of it four times its diameter, nearly filling one of these bulbs with water, exhausting the tube of air by boiling the water, and hermetically sealing it like a thermometer, and afterwards plunging the bulb into a mixture of salt and snow, ice would be immediately formed. This might be repeated as often as wished, in a tube three feet long, when the effects of extracting heat from the water would be very distinct to the eye.

The astronomer royal, Mr. Pond, laid before the society a table of the latitudes of some stars near the north pole, ascertained with great accuracy by means of the newly-erected mural circle, made by Mr. Troughton, and which Mr. P. seems to consider as approaching perfection.

Sir Everard Home furnished a short paper, containing some remarks on the solvent glands and gizzards of the different species of cassowary in New South Wales, and the African ostrich. It appears that these organs are always adapted to the native climate, and to the quantity of food which the fowl can procure. The length of the intestines, and their magnitude, seem dependent on the like cause.

A paper by Mr. Brande, on alcohol, as supplementary to his former remarks on this subject, was read. It appears that Mr. B. has at length succeeded in procuring alcohol from various fermented liquors, without the aid of distillation. For this purpose, he operated with acetate of lead and nitrate of tin, by which he obtained pure alcohol. He also corrected several of his former estimates of the quantity of alcohol in wine, &c.

Dec. 24.—Part of a second paper, by Dr. Lambe, on arsenic, was read. In the account of his experiments, Dr. L. was led to suspect that carbon, azote, and hydrogen, are only modifications of the same element. The society then adjourned till Thursday, Jan. 14, 1813.

---

*Kirwanian Society of Dublin.*—Dec. 16. A memoir on the malic acid was read by M. Donovan, esq.

After making some observations on the combinations of malic acid with lead, the author proceeded to notice certain defects in the processes hitherto proposed for preparing that acid. There is, in the juice of apples, a substance which has heretofore escaped observation, and which possesses very peculiar properties. In the process of Scheele, both are found in what has been supposed to be the resulting pure malic acid; nor can they be separated without the utmost difficulty. In the second process, wherein nitrous acid is distilled from sugar, the author observed that a fluid is produced which does not always manifest in a decisive manner the characteristic properties of malic acid.

The author then adverted to some other defects of these modes of preparing the substance in question, and remarked, that even the process process of Vauquelin, although by much the best, is not free from some of these objections. He then proposed an improvement of the latter process, which affords, as far as is at present known, a malic acid perfectly pure.

The paper concluded with conjectures concerning the state in which this acid, and, perhaps, vegetable acids in general, may previously have existed in fruits and berries. Some experiments and observations were stated, which were supposed to render it probable that the *bitter principle* is a compound basis, which, by uniting to oxygen, or by undergoing more complicated processes, might change its nature so far as to become an acid.

---

*Linnæan Society.*—The last three meetings of this society were chiefly employed in hearing read a monography of the genus *callitriche*, by Mr. Schmaltz.

The paper was in English, of considerable length, and gave a very minute account of this genus, together with an elaborate historical detail of the labors of former botanists, and an ample account of every thing relating to the mode of growth, economy, and uses, of this genus of plants, which, it is well known, grow in water, frequently cover the surface of lakes, and are sometimes so matted together, that, if we believe Linnæus, it is possible to walk over the surface of the water without being wetted. According to Mr. Schmaltz there are six species of callitriche peculiar to the old continent; eight belong to the new; and there are two species found both in the old and new continents.

A paper, by Mr. Sowerby, was likewise read, giving an account of a new cleavage which he had observed in calcareous spar; namely, a cleavage corresponding to the diagonal of the rhomboid, which constitutes the primitive form of calcareous spar.

---

Mr. James Kirkland, surgeon, has in the press an Appendix to an Inquiry into the present State of Medical Surgery, by his father the late Thomas Kirkland, M.D. in which the removal of obstruction and inflammation, in particular instances, with the causes, nature, distinctions, and cure, of Ulcers, is considered; taken from the author's manuscript.

---

Dr. Reid will commence his next course of Lectures on the Practice of Medicine, on Monday, Feb. 15th, at ten o'clock in the morning, at his house, Grenville-street, Brunswick-square.

---

On Thursday, Feb. 4, a course of Lectures will re-commence on the Materia Medica, Practice of Physic, and Chemistry, at eight o'clock in the morning, at No. 9, George-street, Hanover-square, by George Pearson, M.D. F.R.S. senior Physician to St. George's Hospital.

---

*Medical and Chemical Lectures and Examinations.*—Dr. Hooper and Dr. Ager will commence their next course on the Theory and Practice of Physic, Chemistry, and the Materia Medica, on Monday, February 1, 1813, at the Theatre, 26, Cork-street, Burlington Gardens, at eight o'clock in the morning. Three Courses are given yearly, beginning in February, June, and October, each on the first Monday of the month.

